# On Scene Injury Severity Prediction (OSISP) model for trauma developed using the Swedish Trauma Registry

**DOI:** 10.1186/s12911-023-02290-5

**Published:** 2023-10-09

**Authors:** Anna Bakidou, Eva-Corina Caragounis, Magnus Andersson Hagiwara, Anders Jonsson, Bengt Arne Sjöqvist, Stefan Candefjord

**Affiliations:** 1 Department of Electrical Engineering, Chalmers University of Technology, 412 96 Gothenburg, Sweden; 2 Center for Prehospital Research, Faculty of Caring Science, Work Life and Social Welfare, University of Borås, 501 90 Borås, Sweden; 3 Department of Surgery, Institute of Clinical Sciences, Sahlgrenska University Hospital, Sahlgrenska Academy, University of Gothenburg, Per Dubbsgatan 15, 413 45 Gothenburg, Sweden

**Keywords:** Artificial Intelligence (AI), Clinical Decision Support System (CDSS), On Scene Injury Severity Prediction (OSISP), Prehospital care, Trauma, Field triage

## Abstract

**Background:**

Providing optimal care for trauma, the leading cause of death for young adults, remains a challenge e.g., due to field triage limitations in assessing a patient’s condition and deciding on transport destination. Data-driven On Scene Injury Severity Prediction (OSISP) models for motor vehicle crashes have shown potential for providing real-time decision support. The objective of this study is therefore to evaluate if an Artificial Intelligence (AI) based clinical decision support system can identify severely injured trauma patients in the prehospital setting.

**Methods:**

The Swedish Trauma Registry was used to train and validate five models – Logistic Regression, Random Forest, XGBoost, Support Vector Machine and Artificial Neural Network – in a stratified 10-fold cross validation setting and hold-out analysis. The models performed binary classification of the New Injury Severity Score and were evaluated using accuracy metrics, area under the receiver operating characteristic curve (AUC) and Precision-Recall curve (AUCPR), and under- and overtriage rates.

**Results:**

There were 75,602 registrations between 2013–2020 and 47,357 (62.6%) remained after eligibility criteria were applied. Models were based on 21 predictors, including injury location. From the clinical outcome, about 40% of patients were undertriaged and 46% were overtriaged. Models demonstrated potential for improved triaging and yielded AUC between 0.80–0.89 and AUCPR between 0.43–0.62.

**Conclusions:**

AI based OSISP models have potential to provide support during assessment of injury severity. The findings may be used for developing tools to complement field triage protocols, with potential to improve prehospital trauma care and thereby reduce morbidity and mortality for a large patient population.

**Supplementary Information:**

The online version contains supplementary material available at 10.1186/s12911-023-02290-5.

## Background

Trauma is defined as injury caused by external force and covers a wide spectrum of scenarios; penetrating and blunt force trauma; intentional and unintentional trauma; and low- and high-energy trauma, e.g., falls, motor vehicle crashes and violence [1]. It is the leading cause of death in the young population, and accounts for more than 5 million deaths per year globally, corresponding to 9% of the world’s deaths [2]. In addition to its high mortality rate, trauma also represents a high social cost, where in Sweden the cost for injuries is estimated to 60 billion SEK yearly (approximately US$6.4 billion/ €5.8 billion) [3]. Prehospital assessment and care, i.e., care provided at the scene of injury and during transport to a hospital [3], can play a critical role in the delivery of optimal trauma care [4] by facilitating prioritization and deciding adequate destination. Increasing precision in prehospital assessment, prioritization and management of trauma patients is therefore essential to increase personalized care and improve medical outcome.

When arriving at the scene and during transport, the Emergency Medical Services (EMS) clinicians perform field triage to assess severity of injury, prioritize and decide transport destination [5]. If the assessment indicates that a patient is severely injured and has life-threatening conditions, the time to definite optimal care must be minimized to increase the chance of survival [1] – which may not be provided at the closest hospital. To achieve this, the trauma care can be organized with a trauma system that classifies certain medical facilities as trauma centers (TC) depending on their capabilities for managing severely injured patients [6]. The condition of a patient can then be matched with an appropriate destination according to predefined route schemes [6], where direct transportation of severely injured patients to a TC instead of the closest emergency department (ED) reduces the time to definitive care [1] and thereby reduces mortality [7, 8]. Trauma systems have been shown to reduce pooled statistical odds of mortality from 52 studies (OR 0.74, 95% confidence interval 0.69–0.79) [9]. There is no unified trauma system with TC in Sweden [10], a common approach is therefore to approximate university hospitals as TC [11, 12]. By doing so, reduced mortality has also been indicated [11, 13].

Assessment of a patient’s condition is a difficult task that requires both general and individual understanding of the trauma incident to deal with varying circumstances [1]. In Sweden, national trauma team criteria have been recommended for activating a trauma team at an ED [14]. EMS clinicians initiate the procedure and alert the nurse in charge in the ED, who in turn decides the level of trauma team alert. The protocol functions as a checklist and activates a full trauma team (level 1, life-threatening) if any physiologic or anatomic criteria are fulfilled. If none of the physiologic or anatomic criteria have been checked, but any mechanism of injury criteria are fulfilled, a limited trauma team is activated (level 2, potentially life-threatening) [14]. The protocol also contains observation points that may increase the assessed level in case of fulfilled criteria; age < 5 or > 60 years, pregnancy, hypothermia, anticoagulant therapy, serious comorbidity, intoxication or prehospital deterioration [14]. Evaluation of field triage protocols can be performed by assessing the percent of incorrect classifications in terms of undertriage (a severely injured patient being transported to a non-TC), and overtriage (a patient with minor injuries being transported to a TC) [6]. The acceptable level of undertriage is less than 5%, as it has a direct impact on the patient’s chance for survival, whereas overtriage has a higher acceptable range between 25 to 35% as it mostly concerns overcrowding at a TC [6]. In practice, the reported under- and overtriage rates are usually not fulfilling the acceptable levels. High proportions of undertriaged patients have been indicated [11, 15], especially among motor vehicle crashes [12].

The high proportion of undertriaged patients can be understood by realizing the difficult trade-offs made in prehospital decision-making, where the level of resources at the closest hospital is set against the transportation time to a hospital further away with a higher level of resources [1]. In some cases, the results can also be explained by the underestimation of a patient’s care need, which is more common for certain categories of injuries and patients [16]. For instance, age has been identified as an influencing factor for what level of care a patient receives, where elderly with severe injuries are at greater risk of being undertriaged [15–17]. Another example is pre-shock, which can be difficult to detect as particularly children, younger patients and athletes can compensate for a long time before shock is evident [1]. The challenge of reaching acceptable levels of triage accuracy indicates limitations with the current method of assessing a patient’s condition. We believe this may be improved by complementing field triage protocols by making use of mathematics and leverage the potential in statistics and artificial intelligence (AI), e.g., discerning complex patterns between criteria associated with life-threatening conditions. AI based methods for predicting the risk of injury severity can potentially increase precision of trauma severity assessment.

AI can identify complex relationships between variables and has been shown to increase precision in several health care domains. The prehospital care is increasingly represented [18–20], where researchers face challenges with developing models based on incomplete data [21]. Injury severity prediction models for field triage have been studied by Candefjord and associates under the concept of On Scene Injury Severity Prediction (OSISP), focusing on motor vehicle crashes [22–24]. However, the potential of increasing precision in field triage of all trauma incidents in Sweden with AI has not been studied. Prior studies have focused either on subsets of trauma patients (geriatric trauma and motor vehicle crashes), or the complete prehospital patient group, which could be argued to lower the prediction performance due to poor generalization of performance across target domains [25]. Studies on adult trauma have focused on one particular model, a small set of predictors and/or listwise deletion of missing data, which may benefit the performance of simpler models.

The aim of this paper is to evaluate if an AI-based OSISP model for prehospital trauma has potential to complement clinical practice in predicting the risk of injury severity. The models are developed and internally validated with data from the Swedish Trauma Registry (SweTrau) [26]. The long-term ambition is to provide a responsible and explainable [27] data-driven prehospital injury severity classification model for real-time assessment of patients to support prehospital decision making.

## Method

### Source of data

This was a registry study where data from SweTrau, the Swedish national trauma registry, during the period 2013–2020 were used to develop and validate OSISP models. Registration in SweTrau applies to patients fulfilling the following three criteria: 1) All patients where a trauma alert was activated at the hospital; 2) Hospitalized patients with New Injury Severity Score (NISS) > 15, even if they did not trigger a trauma alert; and 3) Patients who were transferred to the hospital within seven days after the traumatic incident and had NISS > 15 [26]. Exclusion criteria apply when the only injury was a chronic subdural hematoma or if a trauma alert was triggered without an underlying traumatic incident [26]. The registrations are managed by each connected hospital via authorized personnel [26].

SweTrau is based on a variable set proposed in the 2008–2009 Utstein protocol, a European consensus protocol for uniform reporting of data following major trauma [28]. The data contains predictive model variables (e.g., age, systolic blood pressure [SBP], dominating type of injury), system characteristic variables (e.g., type of transportation, airway management and highest level of prehospital care provider), and process mapping variables (e.g., timestamps of arrival at scene, first CT scan and first key emergency intervention) related to a patient’s care chain registered at the scene of injury, on arrival at hospital, at discharge and at 30 days after the trauma incident. Injuries are coded retrospectively with the Abbreviated Injury Scale (AIS, version 2005 Update 2008), where a 7-digit code contains information of injury type, location and severity [29]. A multiple injured patient’s overall injury severity status is described using Injury Severity Score (ISS) [30] and New Injury Severity Score (NISS) [31]. ISS is calculated by summing the squares of the three most severe injuries from six predefined body regions [30], whereas NISS is calculated by summing the squares of the three most severe injuries independent of body region [31].

### Sample size

The minimum number of registered trauma incidents needed to develop the prediction model was calculated according to Buderer [32] to decide whether data from SweTrau would be suitable to use. Statistical significance was set as *p* < 0.05 with a tolerance of 1% of the 95% confidence interval (CI). From SweTrau’s annual reports, the prevalence of severely injured patients (NISS > 15) was approximated to 21.3%. To our knowledge, reported clinical practice of undertriage rate and overtriage rate range between 10.5–72.0% and 9.9–48.2%, respectively [15]. With the aim of developing a model that exceeds clinical practice in precision, the expected sensitivity and specificity were set to 90% and 25%, respectively. Calculations showed that a sample size of approximately 16,000 registrations was needed, clearly exceeded by the number of registrations in SweTrau during the selected time-period.

### Participants

According to the annual report of 2020, forty-seven of 49 hospitals providing emergency services (95.9%) in Sweden were connected to SweTrau, where 40 (81.6%) contributed with registrations [33]. The registry had an approximate coverage, i.e. the number of trauma patients with intensive care need in SweTrau compared to the number in the Swedish Intensive Care Registry, of 63.4% in 2020, where the highest amount was obtained from the Stockholm, south and middle healthcare regions and the lowest amounts from the west, north, and southeast healthcare regions [33].

Six sampling exclusion criteria were applied to extract information relevant for field triage in the prehospital setting: 1) Registrations where a prehospital resource was not involved; 2) Transfers between hospitals; 3) Children, i.e. patients younger than 15 years; 4) Data falling outside realistic values according to defined ranges in SweTrau’s manual or as judged by the authors; 5) Duplications, i.e. registrations that shared the same patient ID and where time between trauma incidents was less than 24 h; 6) Missing data in outcome variables.

The definition of a duplication was chosen to enable early readmissions, since multiple readmissions within a year may indicate an increased risk of unplanned readmission of trauma patients [34]. The time difference was calculated with the timestamp for arrival at hospital since it was the day-time variable with least amount of missing data, and instances with a missing date or time were excluded. In the case of a duplication, only the first instance was included in the dataset.

### Predictors

As a first step, potential predictors were chosen based on relevance to injury severity gained from literature [1, 5, 28, 35–38], clinical knowledge and potential to be captured in prehospital settings, resulting in the following set: age, gender, prehospital Glasgow Coma Scale (GCS), motor component of GCS (mGCS), prehospital SBP, prehospital respiratory rate (RR), prehospital cardiac arrest, prehospital airway management and type, season of year, weekday of trauma, time of trauma, time interval between the emergency call and the prehospital resource arriving at the scene (response time), dominating type of injury, mechanism of injury, intention of injury and AIS regions. In SweTrau, the SBP and RR can be registered as continuous (measurements of the vital signs) or categorical (approximations of the vital signs divided into Revised Trauma Score [RTS] levels). Because the data collection is based on different methods, both the continuous and categorical variables for SBP and RR were included.

Potential predictors were included in formats deemed to enable efficient registration in the prehospital setting. Age was dichotomized with 55 years as threshold (≤ 55 and > 55) according to guidelines for field triage [5]. GCS was categorized according to severity of head injuries, i.e., major injury (3–8), moderate (9–12) and mild (13–15) [1]. Motor GCS (mGCS) was defined according to the instrument as 1 (no motor response), 2 (Extension to painful stimuli), 3 (Flexion to painful stimuli), 4 (Withdrawal to painful stimuli), 5 (Localizing painful stimuli), and 6 (Obeys command). SBP RTS was categorized into 0 (0 mmHg), 1 (1–49 mmHg), 2 (50–75 mmHg), 3 (76–89 mmHg), and 4 (> 89 mmHg) and RR RTS into 0 (0 breaths/min), 1 (1–5 breaths/min), 2 (6–9 breaths/min), 3 (> 29 breaths/min), and 4 (10–29 breaths/min). Season, weekday and time were included as they had previously indicated differences in injury characteristics in a German context [35], and similar predictors were generated using SweTrau’s timestamp for the trauma; season of trauma was categorized into spring (March 1 to May 31), summer (June 1 to August 31), autumn (September 1 to November 30) and winter (December 1 to February 28, or 29 in case of a leap year); weekday of trauma was categorized into weekdays (Monday–Sunday); time of trauma was categorized into night (0:00 to 5:59 AM), morning (6:00–11:59 AM), afternoon (12:00 AM–5:59:PM) and evening (6:00–11:59 PM). The response time has shown inconclusive association to trauma outcome in terms of injury severity and mortality [36–38] and was therefore added as a potential predictor, generated by dichotomizing the time difference between SweTrau’s timestamp variables of the alert and arrival at scene into a response time < 8 min or ≥ 8 min [38]. Mechanism of injury was coded according to SweTrau’s definition as Blunt object, Explosion, High energy fall > 3 m, Low energy fall < 3 m, Other, Shot, Stab, Traffic – Bicycle injury, Traffic – Motor vehicle injury, Traffic – Motorcycle injury, Traffic – Pedestrian, Traffic – Other, and Unknown. The AIS regions were generated by extracting body region information from SweTrau’s AIS codes to the following nine binary variables: head, face, neck, thorax, abdomen, spine, upper extremity, lower extremity and external. Remaining predictors were kept according to their definition in SweTrau.

Next, an assessment of each potential predictor’s predictive value for injury severity was performed. A Chi-square univariate test of independence with significance *p* < 0.05 was performed separately on each potential predictor versus the primary outcome (NISS > 15), where Yate’s continuity correction was applied when the degree of freedom was equal to one [39]. The univariate test was also used to select variable in case of similar information, i.e., GCS, mGCS, SBP based on measurements and RTS, and RR based on measurements and RTS. In these cases, the variable with lowest p-value was selected. Logistic regression (LR) was applied for a multivariate analysis of the potential predictors, where variables with statistically significant coefficients were deemed as suitable predictors. Significant result in either of the two statistical tests, i.e., univariate and multivariate, motivated inclusion of the variable in the final set of predictors used to train and validate the machine learning models.

### Machine learning models

Five machine learning techniques were selected based on promising results within prehospital care, emergency medicine, triaging and trauma: LR [19, 20, 40–42], Random Forest (RF) [19, 42–45], Support Vector Machine (SVM) [41, 42], eXtreme Gradient Boosting (XGBoost) [18, 45] and Artificial Neural Networks (ANN) [19, 41, 44]. Because the aim of the study was to explore if there is a potential in using an AI-based OSISP model for prehospital triage of trauma and complement the clinical practice, optimization during the model development was not incorporated in the study design and default settings were used for each model.

A LR model is a supervised learning technique that describes the expected probability of a positive event in terms of a logit function and regression coefficients [46]. Sklearn’s class LogisticRegression was used to implement the model.

A RF model classifies samples by considering the majority vote of several decision trees created from bootstrapped data samples of the original dataset and where the decision trees have been built by randomly considering several of the available variables (with replacements) [47]. Sklearn’s class RandomForestClassifier was used to implement the model.

An XGBoost model performs classification based on the majority vote from several trees, where each tree is created based on residual similarity scores and gains [48]. The model was implemented in Python using the open-source software library XGBoost, with the objective of binary classification. The evaluation metric was set as the area under the precision recall curve (AUCPR) since it has been argued to reflect a model’s performance more accurately in the case of imbalanced data compared to the traditional area under the curve (AUC) for the receiver operating characteristic (ROC) curve [49, 50].

An SVM is a supervised learning technique that transforms data to a higher dimension to find a decision boundary, as a line or hyperplane, which successfully separates classes [51]. Sklearn’s class SVC was used to implement the model.

An ANN consists of a network that resembles the human brain with input units, hidden units and output units and performs nonlinear classification by updating the connections between the units [52]. The model was implemented with Sklearn’s class MLPClassifier.

### Outcome

The NISS was selected as the primary outcome variable of this study because of its wide use in injury severity scoring and accessibility in SweTrau. To assess the sensitivity of the model’s predictive ability in relation to injury severity, the ISS was also used as an outcome measure. Historically, NISS is the successor of ISS and was developed due to the limitation that ISS does not consider multiple severe injuries within the same body region [31]. Although both NISS [31] and ISS [53] correlate with mortality, comparative studies of the two scales reports better predictive power in terms of survival after severe trauma with NISS as compared to ISS [31, 54, 55].

Traditionally, ISS > 15 has been used to define severely injured patients [56], but adjustments of the AIS coding of injuries have led to recommendations of adapting ISS > 12 to decrease the risk of excluding severely injured patients [53]. In the present study, a threshold of 15 was used as definition of severely injured trauma patients. The model’s predictive ability in relation to risk group was assessed by comparing the result of this threshold with a threshold equal to 12, for both NISS and ISS.

The secondary objective of this study was to evaluate whether the OSISP models have potential to complement clinical practice, i.e. whether OSISP has potential to lower field under- and overtriage. Because AIS codes are registered retrospectively at the hospital, the NISS and ISS scores are not available in the prehospital setting and were therefore not suitable as comparison metrics. Instead, under- and overtriage were selected, where undertriage was defined as a severely injured patient being transported to a non-TC and overtriage was defined as a patient with minor injuries transported to a TC, following ACS-COT recommendations [6]. The mapping of hospital name, hospital code and binary classification (TC/non-TC) followed an earlier study [11]. For clinical practice, under- and overtriage was calculated based on the NISS/ISS score registered at the hospital and whether the decided destination was a TC or non-TC. For models, under- and overtriage were calculated based on the predicted NISS/ISS score and an automatic decision of destination based on the NISS/ISS score. The difference in calculations of clinical outcome and models was applied since information about geographic location of the scene of injury was not registered in SweTrau, and therefore a decision of transportation destination could not take the proximity of different hospitals into account.

### Missing data

The high proportion of missing values in trauma registries [21] requires careful consideration to attain a dataset that both represents the population and is sufficiently large for model development. Mainly four approaches for handling missing data in trauma registry-based studies are used: complete case (CC) analysis, subgroup analysis of unknown, multiple imputation (MI) or a combination of CC and MI [57]. The key of selecting a suitable method relies on a realistic assumption of the missing mechanism. In the case of trauma, missing completely at random is generally not a valid assumption [57, 58]. In addition, the missing mechanism in trauma data may vary across variables and registering units, it has therefore been suggested that a more realistic assumption is missing at random (MAR) or a combination of MAR and missing not at random [59].

To our knowledge, the missing mechanisms in SweTrau remains unstudied. We therefore included different approaches to enable a comparison of model performance. From the raw data, instances with missing values in administrative and outcome variables (patient id, time-date variable, ISS, NISS, 30-day mortality, hospital) were removed. Next, four datasets were generated: one based on CC analysis, and three based on different imputation techniques.

In dataset A, CC analysis was applied by examination of different thresholds of missing data in predictors and the effect on data size after listwise deletion. The thresholds ranged from 0 to 100% with an increase of 5%, resulting in 21 datapoints. For each threshold, variables with a larger proportion of missing data were removed and listwise deletion was applied on the remaining predictors. The number of registrations left after the listwise deletion together with the number of remaining predictors were compared to find a threshold that enabled most predictors and instances to be included in dataset A. With this approach, the threshold of acceptable level of missing data was selected to 15%.

Datasets B and C were generated using different imputation techniques on the predictors in dataset A. In dataset B, missing data (missing and unknown) represented a new level in each predictor. In dataset C, a single imputation technique was used to substitute missing values in prehospital predictors representing SBP, GCS and RR with corresponding in-hospital values.

In dataset D, MI was used as it is recommended in the case of MAR [21, 60] and has shown added value in analysis for both prehospital and in-hospital trauma data [59, 61, 62]. More specifically, MI by chained equations (MICE) was applied since it is recommended for non-monotonic missing data [60] and has been used in trauma registry-based studies [63, 64]. Five datasets were imputed where the final set of predictors and the primary outcome (NISS > 15) were used to predict values for the missing locations. The predictors and outcome were in raw format to reduce risk of information loss during imputation. Different imputation methods were applied depending on data type, where numeric data was imputed using predictive mean matching, binary data imputed using a logistic regression model, nominal data imputed using a multinomial logit model, and ordinal data imputed using an ordered logit model [65]. A roman visit schedule and 20 iterations were applied during each imputation procedure.

### Statistical analysis methods

The raw data were used to generate variables of interest and then the exclusion criteria were applied. Next, the described imputation techniques were used to create datasets A–D. The univariate and multivariate tests for selection of predictors were performed on dataset A (CC analysis). Next, the final set of predictors were one-hot encoded to enable numeric input to the machine learning models, and a reference level was selected for each predictor to avoid multicollinearity. Model assessment [52] was performed with a stratified 10-fold cross-validation [66] on dataset A–D. Model evaluation metrics were selected to capture the performance in relation to clinical practice and imbalanced data [67] and included the following: under- and overtriage, accuracy, F-measure with β = 1, ROC curves with the True Positive Rate (TPR) versus False Positive Rate (FPR), AUC, Precision-Recall (ROCPR) Curves with precision versus recall/sensitivity and AUCPR. The cross-validated ROC, ROCPR and F1 scores were based on concatenated – i.e., combined data across the ten folds – true positives, false positives, false negatives and true positives across the folders, while the accuracy, under- and overtriage, AUC and AUCPR were averaged across the folders [68]. Note that the TPR can be interpreted as 1-undertriage, FPR/Recall as overtriage, and precision as the number of patients in need of going to a TC of those that did. The same evaluation metrics were applied on dataset D and were based on the concatenated data from across the folds for each of the imputed datasets (D1–D5).

### Hold-out analysis

In addition to 10-fold cross-validation, a hold-out analysis was performed on dataset A (CC) to evaluate impact on model performance. In the SweTrau data, registrations from year 2020 were included, the first year of the COVID-19 pandemic, which may affect characteristics of injuries [69]. Two cases were tested. In case 1, models were trained on data between 2013–2019 and evaluated on data from 2020. In case 2, models were trained on data between 2013–2015 and 2017–2020 and evaluated on data from 2016. The same dependent variable (NISS > 15), set of predictors and evaluation metrics as for the 10-fold cross-validation were used.

### Software

The analysis was executed in Python version 3.8.5 using packages from Scikit-learn version 1.0.2 (SelectFromModel, LogisticRegression, RandomForestClassifier, SVC, MLPClassifier, StratifiedKFold, accuracy score, f1 score, roc curve, roc auc score, precision recall curve, average precision score), SciPy version 1.5.3 (chi2 contingency), Statsmodels version 0.13.2 (Logit), and the open-source software XGBoost, version 1.5.1. The MICE imputation was implemented using the R package mice version 3.14.0 via rpy2 version 3.4.5. Default settings were used when training and validating models and an additional file presents parameter details (see Table S1 in Additional file 1).

### Ethical considerations

The study was accepted by the Swedish Ethical Review Authority on the 10th of February 2021 (reference number 2020–06899) and conducted in agreement with the ethical references of the Swedish Research Council. All registry data were pseudonymized and the dataset did not contain any personal data. SweTrau data used in this study cannot be made publicly available.

## Results

### Participants

**Fig. 1:**
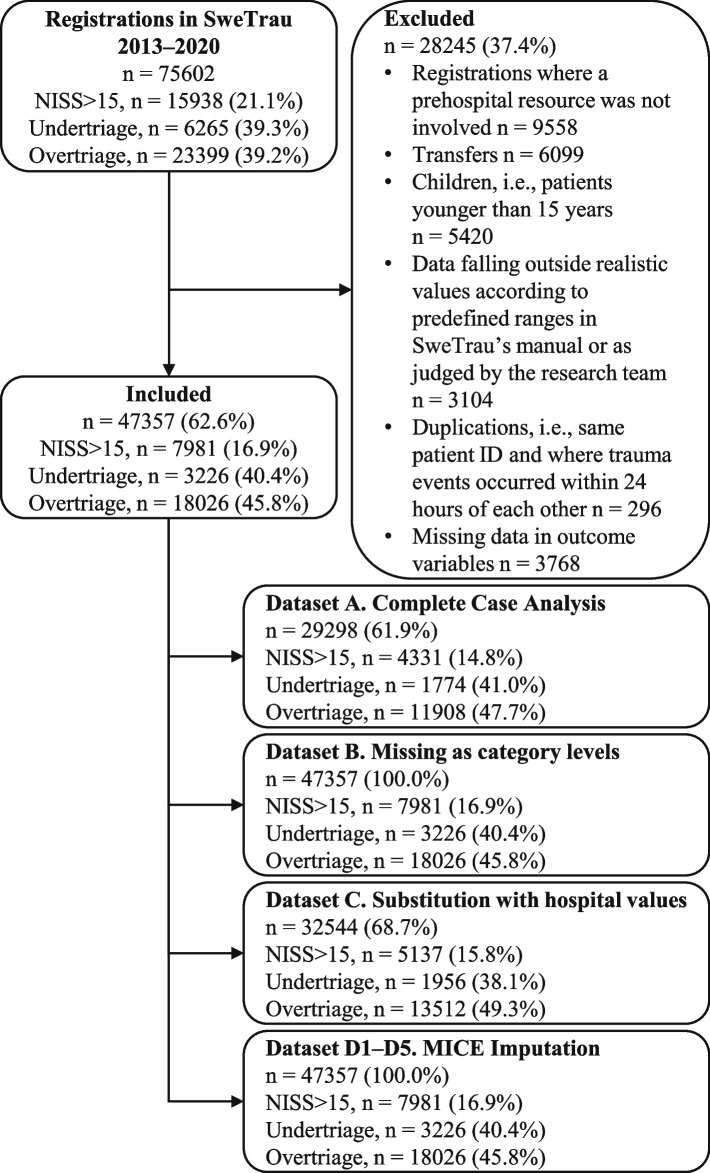
Flow-chart of patient selection with the number of severely injured patients (NISS > 15) and field under- and overtriage included for each step. Instances presented as numbers of cases with percentage in parenthesis

There were 75,602 registrations during the period 2013–2020 and distribution of trauma incidents with respect to year is presented in Table 1. After applying the eligibility criteria 47,357 (62.6%) registrations remained. The patient selection process is displayed in Fig. 1.

**Table 1:** Distribution of trauma incidents with respect to year in raw and included data, presented as number and percentage

Data	Year	Registrations	30-day m	NISS > 15	Undertriage	Overtriage
Raw	2013	7312 (9.7)	246 (3.4)	1401 (19.2)	407 (29.1)	2958 (50.0)
	2014	7754 (10.3)	302 (3.9)	1537 (19.8)	490 (31.9)	3336 (53.7)
	2015	10940 (14.5)	325 (3.0)	1654 (15.1)	495 (29.9)	3869 (41.7)
	2016	11416 (15.1)	350 (3.1)	1753 (15.4)	727 (41.5)	3199 (33.1)
	2017	9846 (13.0)	405 (4.1)	1956 (19.9)	837 (42.8)	2620 (33.2)
	2018	9510 (12.6)	459 (4.8)	2322 (24.4)	939 (40.4)	2806 (39.0)
	2019	9830 (13.0)	486 (4.9)	2708 (27.5)	1157 (42.7)	2395 (33.6)
	2020	8994 (11.9)	470 (5.2)	2607 (29.0)	1213 (46.5)	2216 (34.7)
	Total	75602 (100.0)	3043 (4.0)	15938 (21.1)	6265 (39.3)	23399 (39.2)
Included	2013	4276 (9.0)	103 (2.4)	670 (15.7)	163 (3.8)	2113 (49.4)
	2014	4986 (10.5)	152 (3.1)	761 (15.3)	198 (4.0)	2681 (53.8)
	2015	5591 (11.8)	167 (3.0)	849 (15.2)	232 (4.2)	2888 (51.7)
	2016	6720 (14.2)	204 (3.0)	869 (12.9)	382 (5.7)	2385 (35.5)
	2017	6417 (13.6)	220 (3.4)	988 (15.4)	445 (6.9)	2161 (33.7)
	2018	6467 (13.7)	281 (4.4)	1148 (17.8)	455 (7.0)	2219 (34.3)
	2019	6794 (14.4)	285 (4.2)	1387 (20.4)	647 (9.5)	1943 (28.6)
	2020	6106 (12.9)	272 (4.5)	1309 (21.4)	704 (11.5)	1636 (26.8)
	Total	47357 (62.6)	1684 (3.6)	7981 (16.9)	3226 (40.4)	18026 (45.8)

Distribution of trauma incidents with respect to year in raw and included data, presented as number and percentage. Percentages for 30-day mortality and NISS > 15 are based on the number of registrations per year, percentages for under- and overtriage are based on the number of severely injured and not-severely injured per year. *m* mortality

For case 1 of the hold-out analysis, training data included 25,292 registrations (14.1% severely injured, 38.1% undertriaged, 49.4% overtriaged), and test data included 4006 registrations (19.0% severely injured, 54.3% undertriaged, 36.1% overtriaged). For case 2, training data included 25,027 (15.4% severely injured, 41.1% undertriaged, 48.2% overtriaged), and test data included 4271 registrations (11.1% severely injured, 39.5% undertriaged, 45.1% overtriaged).

### Model development

The threshold of acceptable level of missing data in each variable for construction of Dataset A resulted in a set of possible predictors including gender, age, prehospital GCS and mGCS, prehospital SBP, prehospital RR, prehospital cardiac arrest, prehospital airway management, season of trauma, weekday of trauma, time of trauma, dominating type of injury, mechanism of injury, intention of injury, response time and all AIS regions. In-hospital RR predictors were removed due to a larger amount of missing data than the selected threshold and were therefore not used to substitute missing values in prehospital counterparts in Dataset C.

The univariate and multivariate tests resulted in statistically significant results for different variables. From the univariate analysis, gender, age, airway management, prehospital GCS and mGCS, prehospital SBP, prehospital RR, cardiac arrest, season of year, dominating type of injury, mechanism of injury, response time, and all AIS regions were significant. The mGCS had a lower p-value compared to the GCS and was therefore kept as predictor. From the multivariate analysis, gender, age, mGCS, SBP, RR, injury mechanism, intention of injury and all AIS regions except external had statistically significant coefficients. The coefficients from the multivariate analysis is presented in an additional file (see Table S2 in Additional file 2). Variables that didn’t achieve statistical significance in either of the two tests were weekday and time of trauma and were therefore excluded as predictors. Descriptive statistics of the final set of predictors are presented in Table 2 for the excluded data and dataset A–D.

**Table 2:** Descriptive statistics of study population

Variable	Level	Excluded	Dataset A	Dataset B	Dataset C	Dataset D
Age^a,b^	≤ 55 (Young)^c^	21837 (77.3)	20120 (68.7)	32427 (68.5)	22334 (68.6)	32438 ± 1.3 (68.5)
	> 55 (Elderly)	6342 (22.5)	9178 (31.3)	14914 (31.5)	10210 (31.4)	14919 ± 1.3 (31.5)
	Unknown	66 (0.2)	0 (0)	16 (0.0)	0 (0)	0 ± 0.0 (0.0)
Airway management^a^	Yes	1406 (5.0)	66 (0.2)	361 (0.8)	73 (0.2)	379 ± 1.5 (0.8)
	No^c^	15813 (56.0)	29232 (99.8)	46111 (97.4)	32471 (99.8)	46978 ± 1.5 (99.2)
	Unknown	11026 (39.0)	0 (0)	885 (1.9)	0 (0)	0 ± 0.0 (0.0)
AIS region: Abdomen^a,b^	Yes	2937 (10.4)	2476 (8.5)	4103 (8.7)	2845 (8.7)	4103 ± 0.0 (8.7)
	No^c^	25308 (89.6)	26822 (91.5)	43254 (91.3)	29699 (91.3)	43254 ± 0.0 (91.3)
AIS region: External^a^	Yes	5605 (19.8)	6832 (23.3)	12016 (25.4)	7378 (22.7)	12016 ± 0.0 (25.4)
	No^c^	22640 (80.2)	22466 (76.7)	35341 (74.6)	25166 (77.3)	35341 ± 0.0 (74.6)
AIS region: Face^a,b^	Yes	4613 (16.3)	6174 (21.1)	9835 (20.8)	7097 (21.8)	9835 ± 0.0 (20.8)
	No^c^	23632 (83.7)	23124 (78.9)	37522 (79.2)	25447 (78.2)	37522 ± 0.0 (79.2)
AIS region: Head^a,b^	Yes	8036 (28.5)	8358 (28.5)	13547 (28.6)	9517 (29.2)	13547 ± 0.0 (28.6)
	No^c^	20209 (71.5)	20940 (71.5)	33810 (71.4)	23027 (70.8)	33810 ± 0.0 (71.4)
AIS region: Lower extremity^a,b^	Yes	6267 (22.2)	7973 (27.2)	12644 (26.7)	9131 (28.1)	12644 ± 0.0 (26.7)
	No^c^	21978 (77.8)	21325 (72.8)	34713 (73.3)	23413 (71.9)	34713 ± 0.0 (73.3)
AIS region: Neck^a,b^	Yes	922 (3.3)	2153 (7.3)	3111 (6.6)	2531 (7.8)	3111 ± 0.0 (6.6)
	No^c^	27323 (96.7)	27145 (92.7)	44246 (93.4)	30013 (92.2)	44246 ± 0.0 (93.4)
AIS region: Spine^a,b^	Yes	5358 (19.0)	5196 (17.7)	8622 (18.2)	5758 (17.7)	8622 ± 0.0 (18.2)
	No^c^	22887 (81.0)	24102 (82.3)	38735 (81.8)	26786 (82.3)	38735 ± 0.0 (81.8)
AIS region: Thorax^a,b^	Yes	5963 (21.1)	6314 (21.6)	10587 (22.4)	7179 (22.1)	10587 ± 0.0 (22.4)
	No^c^	22282 (78.9)	22984 (78.4)	36770 (77.6)	25365 (77.9)	36770 ± 0.0 (77.6)
AIS region: Upper extremity^a,b^	Yes	6004 (21.3)	8295 (28.3)	13241 (28.0)	9479 (29.1)	13241 ± 0.0 (28.0)
	No^c^	22241 (78.7)	21003 (71.7)	34116 (72.0)	23065 (70.9)	34116 ± 0.0 (72.0)
Cardiac arrest^a^	Yes	621 (2.2)	55 (0.2)	360 (0.8)	59 (0.2)	375 ± 2.4 (0.8)
	No^c^	16620 (58.8)	29243 (99.8)	46174 (97.5)	32485 (99.8)	46982 ± 2.4 (99.2)
	Unknown	11004 (39.0)	0 (0)	823 (1.7)	0 (0)	0 ± 0.0 (0.0)
Dominating type of injury^a^	Blunt^c^	25354 (89.8)	27207 (92.9)	43279 (91.4)	30057 (92.4)	43395 ± 2.2 (91.6)
	Penetrating	1629 (5.8)	2091 (7.1)	3950 (8.3)	2487 (7.6)	3962 ± 2.2 (8.4)
	Unknown	1262 (4.5)	0 (0)	128 (0.3)	0 (0)	0 ± 0.0 (0.0)
mGCS^a,b^	6. Obeys commands/pain response^c^	12163 (43.1)	27546 (94.0)	38494 (81.3)	30462 (93.6)	43762 ± 9.2 (92.4)
	5. Localising pain	581 (2.1)	981 (3.3)	1502 (3.2)	1162 (3.6)	1776 ± 9.6 (3.7)
	4. Withdrawal from pain	269 (1.0)	312 (1.1)	470 (1.0)	361 (1.1)	567 ± 10.0 (1.2)
	3. Flexion to pain (decorticate)	124 (0.4)	76 (0.3)	117 (0.2)	94 (0.3)	138 ± 3.0 (0.3)
	2. Extension to pain (decerebrate)	126 (0.4)	61 (0.2)	108 (0.2)	70 (0.2)	134 ± 3.7 (0.3)
	1. No motor response	970 (3.4)	322 (1.1)	828 (1.7)	395 (1.2)	981 ± 7.9 (2.1)
	Unknown	14012 (49.6)	0 (0)	5838 (12.3)	0 (0)	0 ± 0.0 (0.0)
Gender^a,b^	Female	9619 (34.1)	10701 (36.5)	16858 (35.6)	11787 (36.2)	16863 ± 1.8 (35.6)
	Male^c^	18591 (65.8)	18597 (63.5)	30487 (64.4)	20757 (63.8)	30494 ± 1.8 (64.4)
	Unknown	35 (0.1)	0 (0)	12 (0.0)	0 (0)	0 ± 0.0 (0.0)
Intention of injury^b^	Accident	24198 (85.7)	26103 (89.1)	41155 (86.9)	28766 (88.4)	41415 ± 7.2 (87.5)
	Self-inflicted	935 (3.3)	888 (3.0)	1796 (3.8)	1065 (3.3)	1833 ± 5.5 (3.9)
	Assault	1499 (5.3)	2258 (7.7)	3929 (8.3)	2659 (8.2)	4003 ± 6.1 (8.5)
	Other^c^	48 (0.2)	49 (0.2)	106 (0.2)	54 (0.2)	107 ± 0.7 (0.2)
	Unknown	1565 (5.5)	0 (0)	371 (0.8)	0 (0)	0 ± 0.0 (0.0)
Mechanism of injury^a,b^	Blunt object	1668 (5.9)	1821 (6.2)	2827 (6.0)	2045 (6.3)	2846 ± 6.0 (6.0)
	Explosion	104 (0.4)	49 (0.2)	97 (0.2)	63 (0.2)	98 ± 0.9 (0.2)
	High energy fall > 3 m	6604 (23.4)	5449 (18.6)	8920 (18.8)	6084 (18.7)	8947 ± 4.2 (18.9)
	Low energy fall < 3 m	2451 (8.7)	3703 (12.6)	5860 (12.4)	4135 (12.7)	5897 ± 6.5 (12.5)
	Other^c^	1651 (5.8)	518 (1.8)	1093 (2.3)	564 (1.7)	1095 ± 1.4 (2.3)
	Shot	413 (1.5)	324 (1.1)	644 (1.4)	385 (1.2)	646 ± 0.8 (1.4)
	Stab	1023 (3.6)	1649 (5.6)	3106 (6.6)	1982 (6.1)	3117 ± 3.2 (6.6)
	Traffic—Bicycle injury	2022 (7.2)	2565 (8.8)	3942 (8.3)	2831 (8.7)	3955 ± 1.6 (8.4)
	Traffic—Motor vehicle injury	7266 (25.7)	9478 (32.4)	14663 (31.0)	10251 (31.5)	14690 ± 4.9 (31.0)
	Traffic—Motorcycle injury	2368 (8.4)	2448 (8.4)	3873 (8.2)	2721 (8.4)	3885 ± 1.6 (8.2)
	Traffic—Pedestrian	845 (3.0)	903 (3.1)	1460 (3.1)	1042 (3.2)	1467 ± 2.7 (3.1)
	Traffic—Other	483 (1.7)	391 (1.3)	711 (1.5)	441 (1.4)	713 ± 1.0 (1.5)
	Unknown	1347 (4.8)	0 (0)	161 (0.3)	0 (0)	0 ± 0.0 (0.0)
Respiratory rate^a,b^	RTS 0: 0	54 (0.2)	18 (0.1)	31 (0.1)	19 (0.1)	58 ± 3.0 (0.1)
	RTS 1: 1–5	23 (0.1)	17 (0.1)	35 (0.1)	19 (0.1)	45 ± 2.1 (0.1)
	RTS 2: 6–9	70 (0.2)	51 (0.2)	91 (0.2)	60 (0.2)	104 ± 1.4 (0.2)
	RTS 3: > 29	1206 (4.3)	1403 (4.8)	2204 (4.7)	1706 (5.2)	2526 ± 16.9 (5.3)
	RTS 4: 10-29^c^	12973 (45.9)	27809 (94.9)	40115 (84.7)	30740 (94.5)	44625 ± 17.4 (94.2)
	Unknown	13919 (49.3)	0 (0)	4881 (10.3)	0 (0)	0 ± 0.0 (0.0)
Response time^a^	< 8 min^c^	4249 (15.0)	7515 (25.7)	12038 (25.4)	8520 (26.2)	12270 ± 8.0 (25.9)
	≥ 8 min	12894 (45.7)	21783 (74.3)	34441 (72.7)	24024 (73.8)	35087 ± 8.0 (74.1)
	Unknown	11102 (39.3)	0 (0)	878 (1.9)	0 (0)	0 ± 0.0 (0.0)
Season of year^a^	Winter^c^	6177 (21.9)	6273 (21.4)	10273 (21.7)	7030 (21.6)	10276 ± 1.0 (21.7)
	Spring	7028 (24.9)	6894 (23.5)	11381 (24.0)	7734 (23.8)	11384 ± 1.1 (24.0)
	Summer	8310 (29.4)	8585 (29.3)	13881 (29.3)	9455 (29.1)	13884 ± 1.4 (29.3)
	Autumn	6701 (23.7)	7546 (25.8)	11811 (24.9)	8325 (25.6)	11813 ± 0.7 (24.9)
	Unknown	29 (0.1)	0 (0)	11 (0.0)	0 (0)	0 ± 0.0 (0.0)
Systolic blood pressure^a,b^	RTS 0: 0	196 (0.7)	17 (0.1)	36 (0.1)	19 (0.1)	129 ± 11.0 (0.3)
	RTS 1: 1–49	16 (0.1)	31 (0.1)	41 (0.1)	33 (0.1)	46 ± 1.9 (0.1)
	RTS 2: 50–75	116 (0.4)	168 (0.6)	271 (0.6)	213 (0.7)	316 ± 3.2 (0.7)
	RTS 3: 76–89	243 (0.9)	327 (1.1)	530 (1.1)	378 (1.2)	600 ± 4.0 (1.3)
	RTS 4: > 89^c^	13310 (47.1)	28755 (98.1)	42246 (89.2)	31901 (98.0)	46266 ± 12.5 (97.7)
	Unknown	14364 (50.9)	0 (0)	4233 (8.9)	0 (0)	0 ± 0.0 (0.0)

^a,b^Denote statistically significant results for univariate and multivariate tests, respectively

^c^Shows selected reference level for each predictor. Instances presented as number of cases with percentage in parenthesis. Dataset D presented with average number of cases and standard deviation across the five imputed datasets (D1–D5), percentage in parenthesis

### Model performance

**Fig. 2:**
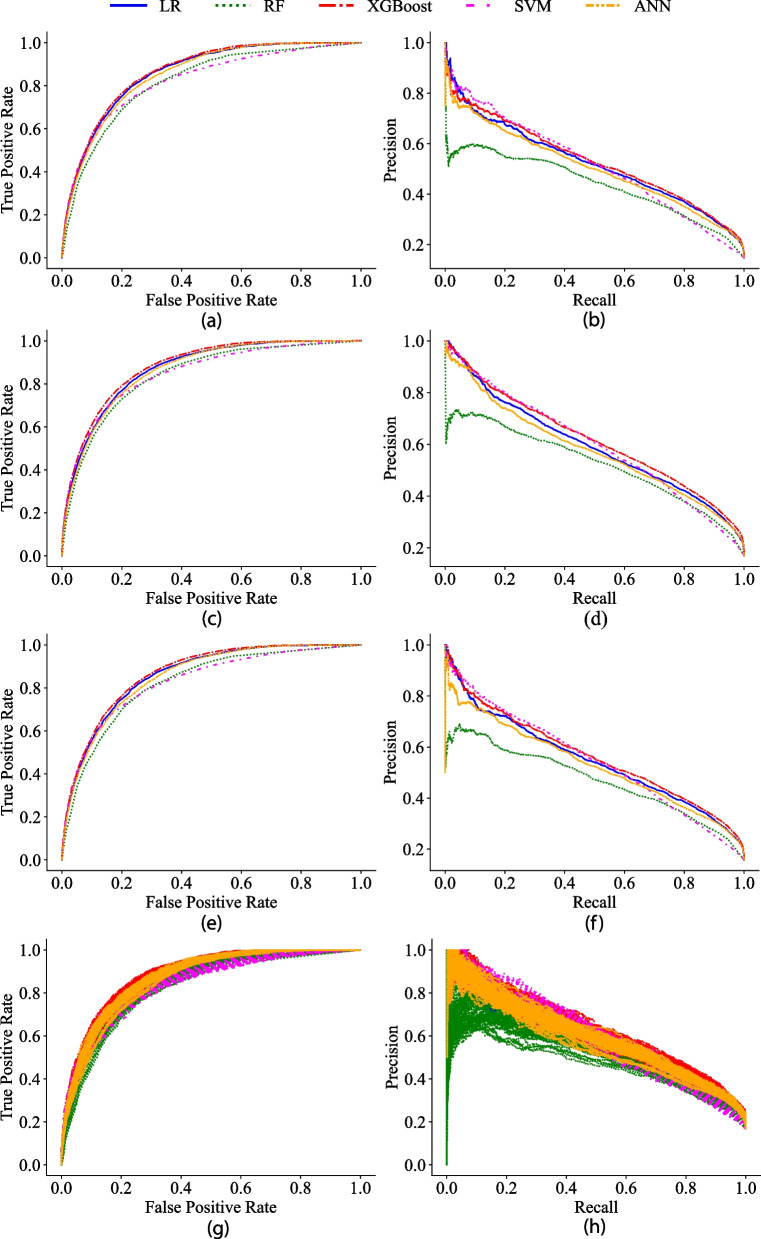
Model performance for predicting the risk of severely injured (NISS > 15). **a**-**b** ROC curve and Precision-Recall curve for dataset A, **c**-**d** ROC curve and Precision-Recall curve for dataset B, **e**–**f**) ROC curve and Precision-Recall curve for dataset C, **g**-**h** ROC curve and Precision-Recall curve for dataset D

Cross-validated ROC and ROCPR curves for all models are visualized in Fig. 2 for each dataset and evaluation metrics are summarized in Table 3. Mapping of under- and overtriage to the ROC curves according to earlier description, the clinical recommendation of 25–35% overtriage yielded an undertriage between 8–25% depending on model and dataset, to be compared with the clinical recommendation of 5%. Reviewing the clinical outcome of field triage in SweTrau for the selected time-period, an undertriage of about 40.4% and overtriage of about 45.8% were obtained. At a corresponding level of overtriage, the cross-validated OSISP models had an undertriage between 4.1–12.4%. To the authors knowledge, there is not a clinical recommendation for precision. The clinical outcome resulted in a precision equal to 20.9% at a recall level of 59.6%. At a corresponding level of recall, the OSISP models had a precision between 41.1–56.4%. Undertriage, overtriage and precision for selected points on the ROC curve according to recommended levels by ACS-COT [6] and an additional point with low undertriage (1%) are presented in Table 4.

**Table 3:** Model performance for predicting the risk of severely injured (NISS > 15)

Metric	Dataset	LR	RF	XGBoost	SVM	ANN
Accuracy [%]	A	86.8 ± 0.33	85.8 ± 0.47	87.0 ± 0.38	87.1 ± 0.33	86.5 ± 0.52
	B	86.1 ± 0.40	85.1 ± 0.64	86.5 ± 0.35	86.5 ± 0.47	85.5 ± 0.55
	C	86.3 ± 0.36	85.1 ± 0.37	86.6 ± 0.42	86.7 ± 0.32	86.0 ± 0.23
	D	86.1–86.2 ± 0.39	85.0–85.2 ± 0.70	86.5–86.6 ± 0.46	86.5–86.6 ± 0.48	85.7–86.0 ± 0.42
F1–score [%]	A	40.0	41.9	43.2	35.9	44.9
	B	47.1	47.9	51.2	46.1	49.8
	C	43.0	43.8	46.2	39.9	46.4
	D	47.4–47.7	48.2–48.6	51.0–51.4	46.8–47.1	50.7–51.2
AUC	A	0.86 ± 0.01	0.82 ± 0.01	0.87 ± 0.01	0.83 ± 0.01	0.85 ± 0.01
	B	0.87 ± 0.01	0.84 ± 0.01	0.88 ± 0.01	0.85 ± 0.01	0.86 ± 0.01
	C	0.86 ± 0.01	0.82 ± 0.01	0.87 ± 0.01	0.83 ± 0.01	0.85 ± 0.01
	D	0.870–0.870 ± 0.01	0.840–0.841 ± 0.01	0.880–0.882 ± 0.01	0.847–0.850 ± 0.01	0.870–0.871 ± 0.01
AUCPR	A	0.52 ± 0.02	0.44 ± 0.02	0.53 ± 0.03	0.52 ± 0.02	0.51 ± 0.02
	B	0.59 ± 0.02	0.52 ± 0.03	0.62 ± 0.02	0.60 ± 0.02	0.58 ± 0.02
	C	0.55 ± 0.02	0.47 ± 0.01	0.57 ± 0.02	0.55 ± 0.02	0.53 ± 0.02
	D	0.595–0.597 ± 0.02	0.517–0.522 ± 0.03	0.615–0.620 ± 0.02	0.59–0.60 ± 0.02	0.589–0.591 ± 0.02

Accuracy, AUC and AUCPR presented as average value and standard deviation across the folds. F1-score presented as concatenated value across all folds. Dataset D presented with an interval of respective value across all folds for the five imputed datasets, with the highest standard deviation

**Table 4:** Model performance for different points on the ROC curve when predicting the risk of severely injured (NISS > 15)

Model			LR				RF				XGBoost				SVM				ANN	
Dataset	A	B	C	D	A	B	C	D	A	B	C	D	A	B	C	D	A	B	C	D
Undertriage^a^	1	1	1	1	1	1	1	1	1	1	1	1	1	1	1	1	1	1	1	1
Overtriage	67	67	67	66–66	1	1	1	1–1	64	60	64	59–60	90	86	91	85–86	66	65	68	63–64
Precision	20	22	22	23–23	15	17	16	17–17	21	25	23	25–25	16	19	17	19–19	21	24	22	24–24
Undertriage^a^	5	5	5	5	5	5	5	5	5	5	5	5	5	5	5	5	5	5	5	5
Overtriage	49	47	49	47–48	1	55	1	54–55	46	43	45	43–44	70	61	66	60–62	49	47	50	45–47
Precision	25	29	27	28–29	15	26	16	26–26	26	31	28	31–31	19	24	21	24–24	25	29	26	29–30
Undertriage	11	10	11	10–10	17	13	16	14–14	10	8	10	8–9	17	14	17	15–15	13	11	12	10–11
Overtriage^a^	35	35	35	35	35	35	35	35	35	35	35	35	35	35	35	35	35	35	35	35
Precision	31	35	33	34–34	29	33	31	33–34	30	34	33	34–35	29	33	31	33–33	30	34	32	34–35
Undertriage	19	17	19	17–17	25	22	24	22–22	18	16	18	16–16	24	21	24	22–22	21	19	21	18–18
Overtriage^a^	25	25	25	25	25	25	25	25	25	25	25	25	25	25	25	25	25	25	25	25
Precision	36	40	38	40–40	34	39	36	39–39	37	41	38	41–41	34	39	36	39–39	36	40	37	39–40

Values are presented as percentage and ^a^denotes fixed metric values. Dataset D presented with an interval across the five imputed datasets (D1–D5)

The ROC curves showed similar performance between the models, where LR, XGBoost and ANN yielded best performance. The ROCPR curves demonstrated better performance than baseline (prevalence in dataset), with LR, XGBoost, SVM and ANN performing similarly, whereas RF yielded the lowest accuracy. Comparison of the ROC and ROCPR curves showed a noisier behavior in the latter in case of low recall.

From Table 3, SVM achieved the highest accuracy while XGBoost performed best in terms of AUC and AUCPR across all datasets. The difference between models were nonetheless minor. Inconclusive results were indicated for the concatenated F1 score as no model performed best across all datasets. ROC and ROCPR curves for each of ISS > 12, ISS > 15 and NISS > 12 as definitions for a severely injured patient performed similarly and AUC and AUCPR are presented in an additional file (see Table S3 in Additional file 3). Removal of AIS regions as predictors resulted in a decline in performance with average AUC respective AUCPR values across the models between 0.57–0.74 and 0.25–0.40. Model performance for the hold-out analysis is presented in Table 5.

**Table 5:** Model performance for predicting the risk of severely injured (NISS > 15) in the hold-out analysis

Metric	Case	LR	RF	XGBoost	SVM	ANN
Accuracy	1	83	83	84	84	83
	2	91	89	91	91	89
F1-Score	1	41	43	42	34	48
	2	44	41	46	39	43
AUC	1	0.83	0.80	0.84	0.81	0.83
	2	0.89	0.85	0.89	0.85	0.87
AUCPR	1	0.54	0.47	0.54	0.54	0.53
	2	0.54	0.43	0.53	0.53	0.46

## Discussion

### Key results

In this study, an OSISP model for adult trauma was developed based on data from SweTrau. Predictors for severe injury were selected based on statistically significant results from univariate and multivariate tests, resulting in 21 included predictors. AIS regions constituted nine of these predictors and seem to be strong predictors. Both ROC and ROCPR curves demonstrated promising performance. Cross-validated evaluation metrics showed similar results across the models and the four different datasets derived from different strategies for handling missing data.

### Limitations

There are several limitations connected to the data source. Data points from SweTrau originate from different hospitals, settings and regions. The number of active hospitals connected to the registry varies across the years, which can lead to a biased representation of hospitals with high level of administrative resources to manage the time-consuming task of registering in quality registries. The eligibility criteria of the registry may disregard some trauma patients cared for by prehospital resources. For example, patients who are declared dead upon arrival at the hospital are not included in the registry. The registration in SweTrau is performed manually by a register nurse at each connected hospital. The data are in different electronic health records and require subjective assessment in some cases. The work requires many resources, e.g. the mean time of registering a patient at Sahlgrenska University Hospital is estimated to about 45 min. There were also some data quality issues, for instance some data falling outside realistic values that had to be discarded.

This study is limited to NISS and ISS as outcome measurements, scales that are similar but with the difference of in which body regions the three most severe injuries of a patient can be located. In future studies, the prediction models’ performance could be further compared with injury severity scales calculated differently from ISS and NISS. In this study, we worked with a binary classification model (not severely injured/severely injured) and binary transport destination (NTC/TC). Multiple classification might be more suitable depending on the destination definition. For instance, in the definition of TC used in the US, each TC is assigned a rating (I, II, III or IV) depending on the level of resources, where rating I represents the highest level and IV the lowest level [6]. Although there is no unified trauma system in Sweden, a similar rank-approach could possibly be adapted, for instance by assigning highest rank to university hospitals, second rank to county hospitals or trauma receiving hospitals, and third rank to remaining non-trauma receiving hospitals. These ratings could then be used as basis for possible destinations, and future models could potentially categorize what risk interval a patient is in and match it with an appropriately ranked hospital. Alternatively, the care needed could also act as a destination selection. For instance, based on the predicted injury severity and locations of injuries a treatment might be recommended and the transport destination could then be based on what hospitals offer that treatment.

The estimated under- and overtriage for the AI models could not take into account the transportation times to the different nearby hospitals. The model performance might therefore be overestimated, as geographical information about nearby hospitals was not accessible and sometimes the transportation time to a TC may be too long to be recommended. Following the same reasoning, the clinical outcome in field triage could be argued to be underestimated as no TCs might have been located within a reasonable time-frame.

This was an exploratory study to evaluate AI-based field triage for the whole adult trauma population group. A more complex approach for optimizing the algorithms used was therefore out of scope but could be considered in future studies. From Tables 3 and 4, the generated datasets seem to have minor impacts on model performance. For dataset A accuracy and precision increase, whereas for dataset D F1-score increases, and for datasets B and D AUC, AUCPR and under- and overtriage increase. The differences are however small and in general the results are in relatively close agreement. An important aspect to consider during model development is data leakage, i.e., when information related to the test set leaks into the training set, which removes the purpose of having a test dataset as it should consist of unseen data and might lead to overoptimistic results [70]. Alternatives for upcoming studies regarding data leakage could benefit from adjusting the predictor selection. In this study, it is based on univariate and multivariate statistical tests based on Dataset A before applying 10-fold cross validation. Another approach could instead be to first divide the included data into ten folders for cross validation, create dataset A–D within each folder and train the model on the current combination of nine folders. Optimization of the models’ hyperparameters may also increase the prediction ability. For instance, selecting a linear kernel for a SVM model in the case of non-linear data will lead to poor performance. Another possibility for optimization is to incorporate techniques developed for imbalanced data [67, 71].

### Interpretation

The results indicate that OSISP has potential to provide effective decision support for EMS clinicians. Injury locations based on AIS coding seem to be strong predictors, also indicated by other studies [20]. There may be some body regions that are stronger predictors, for instance the logistic regression coefficient for the external region was not significant and might be a weaker predictor compared to other body regions. It should be repeated that AIS codes are retrospectively coded at the hospital and not possible to obtain in real-time within the prehospital setting. However, the field triage protocols in some Swedish regions include markings of injury location that can be used to obtain similar information. Data collection of these markings may result in a different model performance as they will be coded in real-time with no time for controlling entered values. Nonetheless, the impact on model performance motivates further studies.

In general, the origin of each variable used to construct a prediction model is important to consider and how it fits in the prehospital trauma workflow. If the model is to function dynamically during the entire workflow the predictors must be readily available. One example in SweTrau is the two prehospital variables for SBP. One contains exact measurements; however, it is generally not recommended to perform exact measurements on-site with consideration to time-sensitive conditions [1]. The other contains approximations more in line with general practice, where the most suitable option of obtaining an approximation is chosen. This may on the other hand bias the data as all variable levels are not considered, only the most suitable from an accessibility perspective, leading to difficult decisions within the data analysis and interpretation of variables during model development. Furthermore, there is no time-point in SweTrau for when the prehospital variables were registered, impeding development of dynamical algorithm development.

The model performance for AI based field triaging in this study shows potential in improving precision and motivates further work towards clinical implementation, since early identification and transport of severely injured patients to a TC potentially improves patients’ outcome both globally [7, 9] and in Sweden [11]. However, when considering how the OSISP algorithm will function in practice the field triage depends on more factors than injury severity scores. The real-time assessment of the patient being severely injured or not will provide a basis for the decision-making of transport destination, but this will also be influenced by factors like distance to the nearest hospital, the nearest hospital’s resources and distance to the TC. Distance to a TC has an impact on patient outcome where mortality is increased with distance [72]. There is a difference between triaging to a TC and bypassing the nearest hospital in a big city compared to the same decision in a rural area where there is a long distance between the hospitals. In Sweden, the likelihood of being transported to a TC is reduced for every kilometer of distance to the center [12]. This does not mean that the patient is triaged incorrectly, as there are large risks in transporting severely injured patients in an ambulance with little opportunity for advanced treatment. For example, according to the authors’ experiences, it can sometimes be the right decision to drive to the local hospital with a patient with uncontrolled bleeding to stabilize the circulation with, for example, blood products. Patients with isolated head injuries may also need to be anesthetized and intubated before a longer transport to a TC. However, these are difficult and complex decisions where EMS clinicians need further support. It is possible, for example, that in addition to an AI-based decision support the possibility of consulting the on-call trauma surgeon at the TC via video for support in transport decisions could bring further improvements to the prehospital workflow.

An important aspect when evaluating model performance is potential implications on clinical outcome. Clinical implementation requires determining an optimal point on the ROC curve. This is however not a trivial task as it should be decided based on both a technical and medical perspective. Recommended levels of under- and overtriage from ACS-COT could act as a reference but may be challenging to achieve in practice. Table 4 show that performance is generally not on par with the recommended levels of 5% undertriage at less than 35% overtriage. Furthermore, the hold-out analysis indicates a time dependent characteristic in the data with an improved performance when excluding data from year 2016 during the training compared to excluding data from 2020 (Table 5). One hypothesis by the authors is that this may be a result of a stricter triaging policy due to a reduction of resources during the pandemic. Another hypothesis may be that COVID-19 led to a change in injury characteristics.

The general reduction of 28% in undertriage compared to the clinical outcome may benefit around 900 patients in the SweTrau dataset used in this study, i.e. about 112 patients per year. This indicates a potential to achieve a more equal care, although not all those patients may benefit from transport to a TC, e.g. depending on prolonged transportation time and type of injury. Today, patient assessment and care are influenced by different factors such as socioeconomics, ethnicity, age and gender. Two examples are that people in socioeconomically vulnerable areas more often receive inadequate care [73], and elderly with severe trauma are at greater risk of being transported to a hospital with insufficient resources to manage the injuries [9, 16]. Because the OSISP algorithm has been developed based on a data-driven approach, such factors are managed during the training of the models and will not influence the prediction during the patient assessment. In addition, a digital tool does not experience the circumstances that EMS clinicians are exposed to, such as stress and tiredness. However, the support will function together with the EMS clinicians and these factors will still need to be considered in terms of how the variables have been measured and entered to the system. Furthermore, with a digital tool there is an opportunity to develop explainable support systems where the classification of a severely injured patient can be displayed to the EMS clinicians in terms of what variables were important for the prediction. This could give the EMS clinician the possibility to evaluate the patient and relate the OSISP recommendation to their clinical experience. For instance, LR may be a preferred model to test in a clinical setting since the models’ performances were similar and the LR model’s coefficients can be used to derive an explanation to why the patient is predicted to have high or low risk of severe injury. In addition to performance differences also fairness, equality, and explainability should be considered when deciding on which model to develop towards clinical implementation [27].

There are some comparative studies that can help indicate whether the models presented here achieve expected performance. Spangler et al. [18] applied machine learning on regional Swedish prehospital data (not limited to trauma), to develop risk scores for three triage related outcomes, achieving AUC values between 0.66–0.89. Kim et al. [19] used adult prehospital trauma data from the US to predict survival and obtained AUC values between 0.71–0.89. van Rein et al. [20] developed a LR model based on regional adult prehospital data from the Netherlands to predict severely injured (ISS > 15), reporting an AUC value of about 0.82 and an undertriage of about 11% at an overtriage of 50%. Previous studies by Candefjord and colleagues [22–24] developed OSISP models for motor vehicle crashes, reaching AUC values of 0.83 for Swedish data and 0.86 for US data, respectively. The models developed in the present study achieve competitive performances in terms of AUC and under- and overtriage. However, direct comparisons are impeded by variations in trauma system and study designs, i.e., data collection and processing, selected outcomes and development procedures.

### Future research

Development of AI models relies heavily on data, where a larger dataset is preferable. This becomes more important when enabling a larger set of predictors as some predictor levels might be rare. To strengthen results where multiple predictors are included, it should therefore be considered to pool data from different countries. For instance, there are other trauma registries that base their variables on the proposed variables from the Utstein protocol. Pooling data from such registries could provide several opportunities for future work. One example is a pooling of data from different registries, where the extended dataset could be used to increase the size of the development and/or constitute an internal validation dataset. This may increase the model’s ability to generalize the result. A second possibility is to use the data from one registry for development and internal validation of a prediction model, and use data from the second registry to validate the model.

The SweTrau data do not represent all vital signs documented during the prehospital assessment. For instance, pulse, oxygen saturation and heart rate are commonly measured and have proven to contain important information about a patient’s state and could be valuable to include in the decision support. These vitals may be recorded in other registries and a combination of these data could therefore be valuable to increase the data basis for model development.

## Conclusions

An OSISP algorithm for trauma related events aimed for prehospital use shows promising results in aiding care givers in distinguishing between severely injured and non-severely injured patients. This could potentially lower undertriage and reduce mortality. Future model optimization is needed to determine the most suitable model. The results warrant further studies for further development and future implementation and clinical studies of AI based tools to complement current tools for prehospital triage.

### Supplementary Information

**Additional file 1: Table S1.** Model specifications.

**Additional file 2: Table S2.** Multivariate logistic regression coefficients during feature selection.

**Additional file 3: Table S3.** Model performance.

## Data Availability

The raw data that were used in this study are available from the Swedish Trauma Registry (SweTrau), but restrictions apply to the availability of these data, which were used under license for the current study and are not publicly available. For information about SweTrau and access to data, see https://rcsyd.se/swetrau/. For questions about requesting data from this study, contact the corresponding author, AB.

## Abbreviations

AI: Artificial Intelligence
AIS: Abbreviated Injury Scale
ANN: Artificial Neural Networks
AUC: Area under the receiver operating curve
AUCPR: Area under the Precision-Recall curve
C3SE: Chalmers Centre for Computational Science and Engineering
CC: Complete Case
CDSS: Clinical Decision Support System
CI: Confidence Interval
ED: Emergency Departments
EMS: Emergency Medical Services
FPR: False Positive Rate
GCS: Glasgow Coma Scale
ISS: Injury Severity Score
LR: Logistic Regression
MAR: Missing at Random
MI: Multiple Imputation
MICE: Multiple Imputation by Chained Equations
NISS: New Injury Severity Score
NTC: Non-trauma center
OSISP: On Scene Injury Severity Prediction
RF: Random Forest
ROC: Receiver Operating Characteristic
ROCPR: Precision-Recall Curve
RR: Respiratory Rate
RTS: Revised Trauma Score
SBP: Systolic Blood Pressure
SNIC: Swedish National Intrastructure for Computing
SVM: Support Vector Machine
SweTrau: The Swedish Trauma Registry
TC: Trauma Center
TPR: True Positive Rate
XGBoost: EXtreme Gradient Boosting

## References

[1] National Association of Emergency Medical Technicians NAEMT (2020). PHTLS: Prehospital Trauma Life Support.

[2] World Health Organization. Injuries and Violence: the Facts 2014. Geneva: World Health Organization; 2014. Available from: https://apps.who.int/iris/handle/10665/149798. Cited 2022 Nov 17.

[3] Lennquist S (2017). Traumatologi.

[4] Magnusson C, Axelsson C, Nilsson L, Strömsöe A, Munters M, Herlitz J (2018). The final assessment and its association with field assessment in patients who were transported by the emergency medical service. Scand J Trauma Resusc Emerg Med.

[5] Sasser SM, Hunt RC, Faul M, Sugerman D, Pearson WS, Dulski T, et al. Guidelines for field triage of injured patients: Recommendations of the national expert panel on field triage, 2011. Atlanta, GA, USA: Centers for Disease Control and Prevention (CDC); 2012. The Morbidity and Mortality Weekly Report (MMWR) Series: Recommendations and Reports 61(1):1–20. Available from: https://pubmed.ncbi.nlm.nih.gov/22237112/. Cited 2022 Nov 17. 22237112

[6] American College of Surgeons (ACS) (2014). Resources for Optimal Care of the Injured Patient.

[7] MacKenzie EJ, Jurkovich GJ, Frey KP, Scharfstein DO (2006). A national evaluation of the effect of trauma-center care on mortality. N Engl J Med.

[8] Moran CG, Lecky F, Bouamra O, Lawrence T, Edwards A, Woodford M (2018). Changing the system-major trauma patients and their outcomes in the NHS (England) 2008–17. EClinicalMedicine.

[9] Alharbi RJ, Shrestha S, Lewis V, Miller C (2021). The effectiveness of trauma care systems at different stages of development in reducing mortality: a systematic review and meta-analysis. World J Emerg Surg.

[10] Socialstyrelsen. Traumavård vid allvarlig händelse. Stockholm: Socialstyrelsen; 2015. 2015–11–5. Available from: https://www.socialstyrelsen.se/globalassets/sharepoint-dokument/artikelkatalog/ovrigt/2015-11-5.pdf. Cited 2022 Nov 17.

[11] Candefjord S, Asker L, Caragounis EC (2020). Mortality of trauma patients treated at trauma centers compared to non-trauma centers in Sweden: a retrospective study. Eur J Trauma Emerg Surg.

[12] Fagerlind H, Harvey L, Candefjord S, Davidsson J, Brown J (2019). Does injury pattern among major road trauma patients influence prehospital transport decisions regardless of the distance to the nearest trauma centre? - a retrospective study. Scand J Trauma Resusc Emerg Med.

[13] Trivedi DJ, Bass GA, Forssten MP, Scheufler K-M, Olivecrona M, Cao Y (2022). The significance of direct transportation to a trauma center on survival for severe traumatic brain injury. Eur J Trauma Emerg Surg.

[14] Landstingens Ömsesidiga Försäkringsbolag (LÖF). Nationella traumalarmskriterier 2017 – Säker Traumavård. Stockholm, Sweden: Landstingens Ömsesidiga Försäkringsbolag; 2017. Available from: https://lof.se/filer/trauma-broschyr.pdf. Cited 2022 Nov 17.

[15] Lupton JR, Davis-O’Reilly C, Jungbauer RM, Newgard CD, Fallat ME, Brown JB, et al. Under-triage and over-triage using the field triage guidelines for injured patients: A systematic review. Prehosp Emerg Care. 2022;1–8. 10.1080/10903127.2022.2043963. 10.1080/10903127.2022.2043963 35191799

[16] Nakahara S, Matsuoka T, Ueno M, Mizushima Y, Ichikawa M, Yokota J (2010). Predictive factors for undertriage among severe blunt trauma patients: what enables them to slip through an established trauma triage protocol?. J Trauma.

[17] Xiang H, Wheeler KK, Groner JI, Shi J, Haley KJ (2014). Undertriage of major trauma patients in the US emergency departments. Am J Emerg Med.

[18] Spangler D, Hermansson T, Smekal D, Blomberg H (2019). A validation of machine learning-based risk scores in the prehospital setting. PLoS One.

[19] Kim D, You S, So S, Lee J, Yook S, Jang DP (2018). A data-driven artificial intelligence model for remote triage in the prehospital environment. PLoS One.

[20] van Rein EAJ, van der Sluijs R, Voskens FJ, Lansink KWW, Houwert RM, Lichtveld RA (2019). Development and validation of a prediction model for prehospital triage of trauma patients. JAMA Surg.

[21] Rue T, Thompson HJ, Rivara FP, Mackenzie EJ, Jurkovich GJ (2008). Managing the common problem of missing data in trauma studies. J Nurs Scholarsh.

[22] Buendia R, Candefjord S, Fagerlind H, Bálint A, Sjöqvist BA (2015). On scene injury severity prediction (OSISP) algorithm for car occupants. Accid Anal Prev.

[23] Candefjord S, Buendia R, Fagerlind H, Bálint A, Wege C, Sjöqvist BA (2015). On-scene injury severity prediction (OSISP) algorithm for truck occupants. Traffic Inj Prev.

[24] Candefjord S, Sheikh Muhammad A, Bangalore P, Buendia R. On scene injury severity prediction (OSISP) machine learning algorithms for motor vehicle crash occupants in US. J Transp Heal 202;22:101124 10.1016/j.jth.2021.101124.

[25] Liu B. “Weak AI” is likely to never become “strong AI”, so what is its greatest value for us?. arXiv:2103.15294 [cs.Al]. 2021;1–7. 10.48550/arXiv.2103.15294.

[26] The Swedish Trauma Registry (SweTrau). SweTrau | Svenska Traumaregistret. Solna: SweTrau; 2021. Available from: https://rcsyd.se/swetrau/. Cited 2022 Nov 17.

[27] Trocin C, Mikalef P, Papamitsiou Z, Conboy K (2021). Responsible AI for digital health: a synthesis and a research agenda. Inf Syst Front.

[28] Ringdal KG, Coats TJ, Lefering R, Di Bartolomeo S, Steen PA, Røise O (2008). The utstein template for uniform reporting of data following major trauma: a joint revision by SCANTEM, TARN, DGU-TR and RITG. Scand J Trauma Resusc Emerg Med.

[29] Association for the Advancement of Automotive Medicine. Abbreviated Injury Scale (AIS). Chicago: Association for the Advancement of Automotive Medicine; [date unknown]. Available from: https://www.aaam.org/abbreviated-injury-scale-ais/. Cited 2022 Nov 17.

[30] Baker SP, O’Neill B, Haddon WJ, Long WB (1974). The injury severity score: a method for describing patients with multiple injuries and evaluating emergency care. J Trauma.

[31] Osler T, Baker SP, Long W (1997). A modification of the injury severity score that both improves accuracy and scoring. J Trauma.

[32] Buderer NMF (1996). Statistical methodology: I incorporating the prevalence of disease into the sample size calculation for sensitivity and specificity. Acad Emerg Med.

[33] The Swedish Trauma Registry (SweTrau). Årsrapport 2020. Solna: SweTrau; 2021. Årsrapporter; 2020. Available from: https://rcsyd.se/swetrau/wp-content/uploads/sites/10/2021/09/Arsrapport-SweTrau-2020.pdf. Cited 2022 Nov 17.

[34] Moore L, Stelfox HT, Turgeon AF, Nathens AB, Le Sage N, Émond M (2014). Rates, patterns, and determinants of unplanned readmission after traumatic injury: A multicenter cohort study. Ann Surg.

[35] Pape-Köhler CIA, Simanski C, Nienaber U, Lefering R (2014). External factors and the incidence of severe trauma: Time, date, season and moon. Injury.

[36] Bagher A, Todorova L, Andersson L, Wingren C, Ottosson A, Wangefjord S (2017). Analysis of pre-hospital rescue times on mortality in trauma patients in a Scandinavian urban setting. Trauma.

[37] Hosseinzadeh A, Kluger R (2021). Do EMS times associate with injury severity?. Accid Anal Prev.

[38] Blanchard IE, Doig CJ, Hagel BE, Anton AR, Zygun DA, Kortbeek JB (2012). Emergency medical services response time and mortality in an urban setting. Prehosp Emerg Care.

[39] Campbell MJ, Walters SJ, Machin D (2007). Chapter 8, Tests for comparing two groups of categorical or continuous data. Medical Statistics: a Textbook for the Health Sciences.

[40] Suzuki T, Kimura A, Sasaki R, Uemura T (2017). A survival prediction logistic regression models for blunt trauma victims in Japan. Acute Med Surg.

[41] Rau C-S, Wu S-C, Chuang J-F, Huang C-Y, Liu H-T, Chien P-C (2019). Machine learning models of survival prediction in trauma patients. J Clin Med.

[42] Lammers D, Marenco C, Morte K, Conner J, Williams J, Bax T (2022). Machine learning for military trauma: Novel massive transfusion predictive models in combat zones. J Surg Res.

[43] Raita Y, Goto T, Faridi MK, Brown DFM, Camargo CA, Hasegawa K (2019). Emergency department triage prediction of clinical outcomes using machine learning models. Crit Care.

[44] Siebelt M, Das D, Van Den Moosdijk A, Warren T, Van Der Putten P, Van Der Weegen W (2021). Machine learning algorithms trained with pre-hospital acquired history-taking data can accurately differentiate diagnoses in patients with hip complaints. Acta Orthop.

[45] Sánchez-Salmerón R, Gómez-Urquiza JL, Albendín-García L, Correa-Rodríguez M, Martos-Cabrera MB, Velando-Soriano A (2022). Machine learning methods applied to triage in emergency services: A systematic review. Int Emerg Nurs.

[46] Campbell MJ, Walters SJ, Machin D (2007). Chapter 9, Correlation, linear and logistic regression. Medical Statistics: a Textbook for the Health Sciences.

[47] Ho TK. Random decision forests. In: Proceedings of 3rd International Conference on Document Analysis and Recognition. International Conference on Document Analysis and Recognition. August 14–16, 1995; Montrea: IEEE; 1995. p. 278–82. Available from: https://ieeexplore.ieee.org/document/598994. Cited 2022 Nov 17.

[48] Chen T, Guestrin C. XGBoost: A scalable tree boosting system [Internet]. In: Proceedings of the 22nd ACM SIGKDD International Conference on Knowledge Discovery and Data Mining. KDD '16: The 22nd ACM SIGKDD International Conference on Knowledge Discovery and Data Mining. August 13–17, 2016; San Francisco: Association for Computing Machinery; 2016. p. 785–94. Available from: https://dl.acm.org/doi/10.1145/2939672.2939785. Cited 2022 Nov 17.

[49] Saito T, Rehmsmeier M (2015). The precision-recall plot is more informative than the ROC plot when evaluating binary classifiers on imbalanced datasets. PLoS One.

[50] Kaur H, Pannu HS, Malhi AK (2020). A systematic review on imbalanced data challenges in machine learning: Applications and solutions. ACM Comput.

[51] Boser BE, Guyon IM, Vapnik VN. A training algorithm for optimal margin classifiers. In: Proceedings of the Fifth Annual Workshop on Computational Learning Theory. COLT92: 5th Annual Workshop on Computational Learning. July 27–29, 1992; Pittsburgh, Pennsylvani: Association for Computing Machinery; 1992. p. 144–52. Available from: https://dl.acm.org/doi/10.1145/130385.130401.Cited 2022 Nov 17.

[52] Hastie T, Tibshirani R., Friedman, J. The Elements of Statistical Learning. 2nd ed. New York: Springer; 2009. Available from: https://link.springer.com/content/pdf/10.1007/978-0-387-84858-7.pdf. Cited 2022 Nov 17.

[53] Palmer CS, Gabbe BJ, Cameron PA (2016). Defining major trauma using the 2008 abbreviated injury scale. Injury.

[54] Whitaker IY, Gennari TD, Whitaker AL. The difference between ISS and NISS in a series of trauma patients in Brazil. In: 47th Annual Proceedings of the Association for the Advancement of Automotive Medicine. Association for the Advancement of Automotive Medicine 47th Annual Conference. September 22–24, 2003; Lisbon, Portugal. Barrington: Association for the Advancement of Automotive Medicine; 2003. p. 301–9. Available from: https://pubmed.ncbi.nlm.nih.gov/12941232/. Cited 2022 Nov 17. PMC3217565 12941232

[55] Li H, Ma Y-F (2021). New injury severity score (NISS) outperforms injury severity score (ISS) in the evaluation of severe blunt trauma patients. Chin J Traumatol.

[56] Palmer C. Major trauma and the injury severity score - where should we set the bar? In: 51st Annual Proceedings of the Association for the Advancement of Automotive Medicine. Association for the Advancement of Automotive Medicine 47th Annual Conference. October 15–17, 2007; Melbourne: Association for the Advancement of Automotive Medicine; 2007. p. 13–29. Available from: https://pubmed.ncbi.nlm.nih.gov/18184482/. Cited 2022 Nov 17. PMC3217501 18184482

[57] Shivasabesan G, Mitra B, O’Reilly GM (2018). Missing data in trauma registries: A systematic review. Injury.

[58] Roudsari B, Field C, Caetano R (2008). Clustered and missing data in the US national trauma data bank: implications for analysis. Inj Prev.

[59] Moore L, Hanley JA, Lavoie A, Turgeon A (2009). Evaluating the validity of multiple imputation for missing physiological data in the national trauma data bank. J Emerg Trauma Shock.

[60] Jakobsen JC, Gluud C, Wetterslev J, Winkel P (2017). When and how should multiple imputation be used for handling missing data in randomised clinical trials – a practical guide with flowcharts. BMC Med Res Methodol.

[61] O’Reilly GM, Jolley DJ, Cameron PA, Gabbe B (2010). Missing in action: A case study of the application of methods for dealing with missing data to trauma system benchmarking. Acad Emerg Med.

[62] Newgard CD (2006). The validity of using multiple imputation for missing out-of-hospital data in a state trauma registry. Acad Emerg Med.

[63] Glance LG, Osler TM, Mukamel DB, Meredith W, Dick AW (2009). Impact of statistical approaches for handling missing data on trauma center quality. Ann Surg.

[64] Henriksson M, Saulnier DD, Berg J, Gerdin Wärnberg M (2020). The transfer of clinical prediction models for early trauma care had uncertain effects on mistriage. J Clin Epidemiol.

[65] van Buuren S, Groothuis-Oudshoorn K (2011). mice : Multivariate imputation by chained equations in R. J Stat Softw.

[66] Kohavi RA. A study of cross-validation and bootstrap for accuracy estimation and model selection. In: Proceedings of the 14th International Joint Conference on Artificial Intelligence. The 1995 International Joint Conference on AI. August 20–25, 1995; Montreal: Morgan Kaufmann Publishers Inc.; 1995. p. 1137–43. Available from: https://dl.acm.org/doi/10.5555/1643031.1643047. Cited 2022 Nov 17.

[67] He H, Garcia EA (2009). Learning from imbalanced data. IEEE Trans Knowl Data Eng.

[68] Forman G, Scholz M (2010). Apples-to-apples in cross-validation studies: pitfalls in classifier performance measurement. SIGKDD Explor Newsl.

[69] Sherman WF, Khadra HS, Kale NN, Wu VJ, Gladden PB, Lee OC (2021). How did the number and type of injuries in patients presenting to a regional level I trauma center change during the COVID-19 pandemic with a stay-at-home order?. Clin Orthop Relat Res.

[70] Saravanan N, Sathish G, Balajee JM (2018). Data wrangling and data leakage in machine learning for healthcare. J Emerg Technol Innov Res.

[71] Haixiang G, Yijing L, Shang J, Mingyun G, Yuanyue H, Bing G (2017). Learning from class-imbalanced data: Review of methods and applications. Expert Syst Appl.

[72] Wiratama BS, Chen P-L, Chao C-J, Wang M-H, Saleh W, Lin H-A (2021). Effect of distance to trauma centre, trauma centre level, and trauma centre region on fatal injuries among motorcyclists in Taiwan. Int J Environ Res Public Health.

[73] Niklasson A, Herlitz J, Jood K (2019). Socioeconomic disparities in prehospital stroke care. Scand J Trauma Resusc Emerg Med.

