# Supplementary material for: On Scene Injury Severity Prediction (OSISP) model for trauma developed using the Swedish Trauma Registry
Source: BMC Med Inform Decis Mak. 2023 Oct 9;23:206. doi: 10.1186/s12911-023-02290-5 (PMC10561449; doi:10.1186/s12911-023-02290-5)
Supplement: Supplementary file 1 — Additional file 1: Table S1. Model specifications. [file 12911_2023_2290_MOESM1_ESM.docx]

**Additional file 1**

Model specifications

**Table S1.** Model specifications.

| Model | Parameters |
| --- | --- |
| LR | C: 1.0, dual: False, fit intercept: True,  intercept scaling: 1, max iter: 100, multi class:  auto, penalty: l2, solver: lbfgs, tol: 0.0001, verbose:  0, warm start: False |
| RF | C: 1.0, dual: False, fit intercept: True,  intercept scaling: 1, max iter: 100, multi class:  auto, penalty: l2, solver: lbfgs, tol: 0.0001, verbose:  0, warm start: False |
| XGBoost | objective: binary:logistic, use label encoder:True,  enable categorical: False, n estimators: 100, seed:  313 |
| SVM | C: 1, break ties: False, cache size: 200, coef0: 0,  decision function shape: ovr, degree: 3, gamma:  scale, kernel: rbf, max iter: -1, probability: True,  random state: 313, shrinking: True, tol: 0.001, ver-  bose: False |
| ANN | activation: relu, alpha: 0.0001, batch size: auto,  beta 1: 0.9, beta 2: 0.999, early stopping: False,  epsilon: 1e-08, hidden layer sizes: ”(100: )”,  learning rate: constant, learning rate init: 0.001,  max fun: 15000, max iter: 200, momentum: 0.9,  n iter no change: 10, nesterovs momentum: True,  power t: 0.5, random state: 313, shuffle: True,  solver: adam, tol: 0.0001, validation fraction: 0.1,  verbose: False, warm start: False |
