# Supplementary material for: On Scene Injury Severity Prediction (OSISP) model for trauma developed using the Swedish Trauma Registry
Source: BMC Med Inform Decis Mak. 2023 Oct 9;23:206. doi: 10.1186/s12911-023-02290-5 (PMC10561449; doi:10.1186/s12911-023-02290-5)
Supplement: Supplementary file 2 — Additional file 2: Table S2. Multivariate logistic regression coefficients during feature selection. [file 12911_2023_2290_MOESM2_ESM.docx]

**Additional file 2**

Multivariate logistic regression coefficients during feature selection

**Table S2.** Multivariate logistic regression coefficients during feature selection. † denotes statistically significant results.

| Tested predictors | Coefficients |
| --- | --- |
| Age† | 0.6656 |
| Airway management | 0.4771 |
| AIS region: Abdomen† | 0.9299 |
| AIS region: External | 0.0152 |
| AIS region: Face† | 0.2188 |
| AIS region: Head† | 1.2133 |
| AIS region: Lower extremity† | 0.5684 |
| AIS region: Neck† | -0.7999 |
| AIS region: Spine† | 0.9885 |
| AIS region: Thorax† | 1.8705 |
| AIS region: Upper extremity† | 0.3541 |
| Cardiac arrest | -0.5061 |
| Dominating type of injury | 0.1429 |
| GCS (motor) † | -0.4388 |
| Gender† | -0.3091 |
| Intention of injury† | -0.0922 |
| Mechanism of injury† | 0.0669 |
| Respiratory rate† | 0.3116 |
| Response time | -0.0678 |
| Season of year | -0.0006 |
| Systolic blood pressure† | -0.6765 |
| Time of trauma | -0.0087 |
| Weekday of trauma | -0.0080 |
