# Supplementary material for: On Scene Injury Severity Prediction (OSISP) model for trauma developed using the Swedish Trauma Registry
Source: BMC Med Inform Decis Mak. 2023 Oct 9;23:206. doi: 10.1186/s12911-023-02290-5 (PMC10561449; doi:10.1186/s12911-023-02290-5)
Supplement: Supplementary file 3 — Additional file 3: Table S3. Model performance. [file 12911_2023_2290_MOESM3_ESM.docx]

**Additional file 3**

Model performance

**Table S3.** Model performance for predicting the risk of severely injured for different definitions.

| Model | Dataset | | | ISS>12 | | | ISS*>*15 | | | NISS*>*12 | | |
| --- | --- | --- | --- | --- | --- | --- | --- | --- | --- | --- | --- | --- |
|  |  |  |  | AUC | AUCPR | | AUC | AUCPR | | AUC | AUCPR | |
| LR | | A | 0.88 | | | 0.53 | 0.88 | | 0.45 | 0.85 | | 0.56 |
|  | | B | 0.89 | | | 0.6 | 0.89 | | 0.54 | 0.86 | | 0.61 |
|  | | C | 0.88 | | | 0.56 | 0.88 | | 0.49 | 0.85 | | 0.58 |
|  | | D | 0.89-0.89 0.60-0.60 0.89-0.89 0.54-0.55 0.86-0.86 0.62-0.62 | | | | | | | | | |
| RF | | A | 0.85 0.45 0.84 0.35 0.82 0.47 | | | | | | | | | |
|  | | B | 0.87 0.53 0.86 0.46 0.84 0.55 | | | | | | | | | |
|  | | C | 0.85 0.48 0.84 0.39 0.82 0.5 | | | | | | | | | |
|  | | D | 0.86-0.86 0.52-0.53 0.85-0.86 0.46-0.46 0.83-0.83 0.54-0.55 | | | | | | | | | |
| XGBoost | | A | 0.89 0.53 0.89 0.46 0.86 0.56 | | | | | | | | | |
| B | | | 0.9 0.62 | | | | 0.9 0.56 | | | 0.87 0.64 | | |
| C | | | 0.89 0.57 | | | | 0.89 0.49 | | | 0.86 0.59 | | |
|  | | D | 0.90-0.90 0.62-0.62 0.90-0.90 0.56-0.57 0.87-0.87 0.64-0.64 | | | | | | | | | |
| SVM | | A | 0.85 0.52 0.82 0.43 0.83 0.55 | | | | | | | | | |
| B | | | 0.87 0.61 | | | | 0.85 0.54 | | | 0.85 0.62 | | |
| C | | | 0.86 0.56 | | | | 0.83 0.48 | | | 0.83 0.57 | | |
|  | | D | 0.87-0.88 0.61-0.61 0.84-0.84 0.53-0.54 0.85-0.85 0.62-0.62 | | | | | | | | | |
| ANN | | A | 0.88 0.51 0.87 0.42 0.85 0.54 | | | | | | | | | |
| B | | | 0.88 0.58 0.88 0.51 0.86 0.6 | | | | | | | | | |
| C | | | 0.88 0.54 0.87 0.46 0.85 0.56 | | | | | | | | | |
| D | | | 0.89-0.89 0.59-0.59 0.88-0.89 0.52-0.53 0.86-0.86 0.61-0.61 | | | | | | | | | |
| AUC and AUCPR presented as average value across the folds for Dataset A–C, and Dataset D presented with an interval across all folds for the five imputed datasets. | | | | | | | | | | | | |
